# Implementing a family-based intervention to promote healthy family routines in deprived neighborhoods – a feasibility study from Bremen, Germany

**DOI:** 10.1186/s12889-025-25532-9

**Published:** 2025-12-23

**Authors:** Marie Lisanne Schepan, Wolfgang Ahrens, Thomas Altgeld, Heide Busse, Jess Haines, Elisabeth Rataj, Annika Swenne, Maike Wolters, Hajo Zeeb, Tilman Brand

**Affiliations:** 1 Leibniz Institute for Prevention Research and Epidemiology – BIPS, Achterstraße 30, 28359 Bremen, Germany; 2 University of Bremen, Bibliothekstraße 1, Bremen, 28359 Germany; 3 State Association for Health and Academy for Social Medicine Lower Saxony Bremen e. V., Schillerstr. 32, 30159 Hannover, Germany; 4 University of Guelph, 50 Stone Road East, Guelph, ON N1G 2W1 Canada

**Keywords:** Childhood obesity, Family-based interventions, Inequality, Obesity prevention

## Abstract

**Background:**

The prevalence of overweight and obesity among children in Europe is high. Particularly ethnic minority and low-income families are at increased risk. This highlights the need for effective family-based interventions addressing multiple obesity-related behaviors tailored to socially deprived families. Addressing this issue, our study aimed to adapt the North American ‘Healthy Habits, Happy Homes’ program to socially deprived neighborhoods in Bremen, Germany, and to test the feasibility of its implementation by using the RE-AIM model.

**Methods:**

This single-arm intervention study was conducted between 2022 and 2023, including a process evaluation and a pre-post outcome assessment. Families with children aged 3–10 received 4 intervention visits from a health educator over a period of 3–9 months. The implementation fidelity was assessed via an intervention logbook. The acceptability and maintenance of intervention was assessed via feedback questionnaire filled out by parents and through qualitative data collected from parents, intervention staff and local stakeholders. The pre-post outcome assessment was conducted to evaluate its acceptability and to estimate changes in weight status of parents and children, and the children’s dietary habits, physical activity, sleep duration, screen time and behavioral problems.

**Results:**

In total, 23 caregivers and 33 children participated in the intervention study, and 87% of the families reported at least one indicator of social deprivation. Recruitment proved to be challenging; active recruitment methods, i.e. approaching families personally, were more effective than passive ones. The program implementation demonstrated good fidelity and acceptability. Language barriers were perceived as obstacles in program delivery. 78% of the families completed the outcome measurement. Descriptive analysis revealed positive trends over time in increased physical activity, reduced screen time, and behavioral problems, but no changes in BMI, dietary habits, or sleep duration. Stakeholders recommended integrating the intervention into existing structures like kindergartens for long-term implementation.

**Conclusion:**

This study highlights the program’s acceptability and potential benefits for families in deprived neighborhoods of a German city, while acknowledging recruitment challenges. Further adaptations are needed to address barriers and integrate the intervention into wider community efforts to combat childhood obesity.

**Supplementary Information:**

The online version contains supplementary material available at 10.1186/s12889-025-25532-9.

## Introduction

Many European countries are experiencing high levels of childhood overweight and obesity, which can lead to a range of adverse short- and long-term health consequences [1, 2]. Most recent data from Germany shows that 15.4% of children aged 3–17 years in Germany are overweight and 5.9% are obese [3], which is comparable to other European countries [4]. Mounting evidence suggests social and ethnic disparities are associated with a higher risk of childhood overweight and obesity in high-income countries [5, 6]. The reasons why social deprivation and ethnic minority status lead to a higher risk of childhood overweight and obesity are complex and interrelated, including, for example, increased exposure to ultra-processed food advertisement and consumption [7, 8], food insecurity and low levels of food literacy [9–12], poor access to physical activity (PA) facilities and lower levels of vigorous PA [13, 14], unsupervised screen time, and sleep difficulties [15, 16].

A multitude of different interventions aiming at the promotion of PA and a healthy diet in children have been developed and evaluated over the last few decades. The 2019 Cochrane review examined 153 randomized controlled trials (RCTs), highlighting the potential of interventions targeting multiple obesity-related behaviors, rather than individual ones alone [17]. Particularly in the early years, families play an important role in children’s health, and healthy family routines can aid prevent overweight and obesity [18–20].

Several family-based interventions have been tested across various settings [21], yet many of them do not address multiple obesity-related behaviors, with ethnic minorities or migrant families frequently underrepresented [22]. One example of a promising family-based intervention is the ‘Healthy Habits, Happy Homes’ program, originally developed in the US, which aims to promote multiple household routines (e.g. family meals, adequate sleep) using motivational interviewing. Findings of an RCT involving 121 families of mainly African-American and Latino backgrounds showed that children in intervention families decreased their body mass index (BMI), decreased their screen time, and increased their sleep duration [23]. The intervention has since been adapted to the Canadian context; a pilot RCT study demonstrated feasibility, acceptability among Canadian families, and preliminary beneficial effects on children’s dietary intake and obesity levels [24, 25].

Over the last few years, recognition has grown that interventions that have shown efficacy in one place cannot simply be transferred to another due to significant differences in local systems [26, 27]. More research is needed to gain a better understanding of the necessary adaptation steps and procedures for successfully transferring an effective intervention to another context [28, 29]. The objective of this study was to test the feasibility of implementing the adapted ‘Healthy Habits, Happy Homes’ program in socially deprived, ethnically diverse neighborhoods in Bremen, Germany. Specifically, the study aimed (i) to assess the intervention’s reach among socially deprived and ethnically diverse families, (ii) to assess the fidelity, acceptability and perceived effects of the intervention, (iii) to evaluate the acceptability of the outcome evaluation, and (iv) to estimate the change in measures of healthy weight development and behavior change over the course of the study. This feasibility study forms part of the GrowH! project (Growing up healthy: obesity prevention tailored to critical transition periods in the early life-course; https://www.growh.eu), with the overall aim to investigate innovative interventions for establishing and sustaining healthy lifestyles at population level to reverse current trends in childhood overweight and obesity.

## Methods

### Study design

**Fig. 1:**
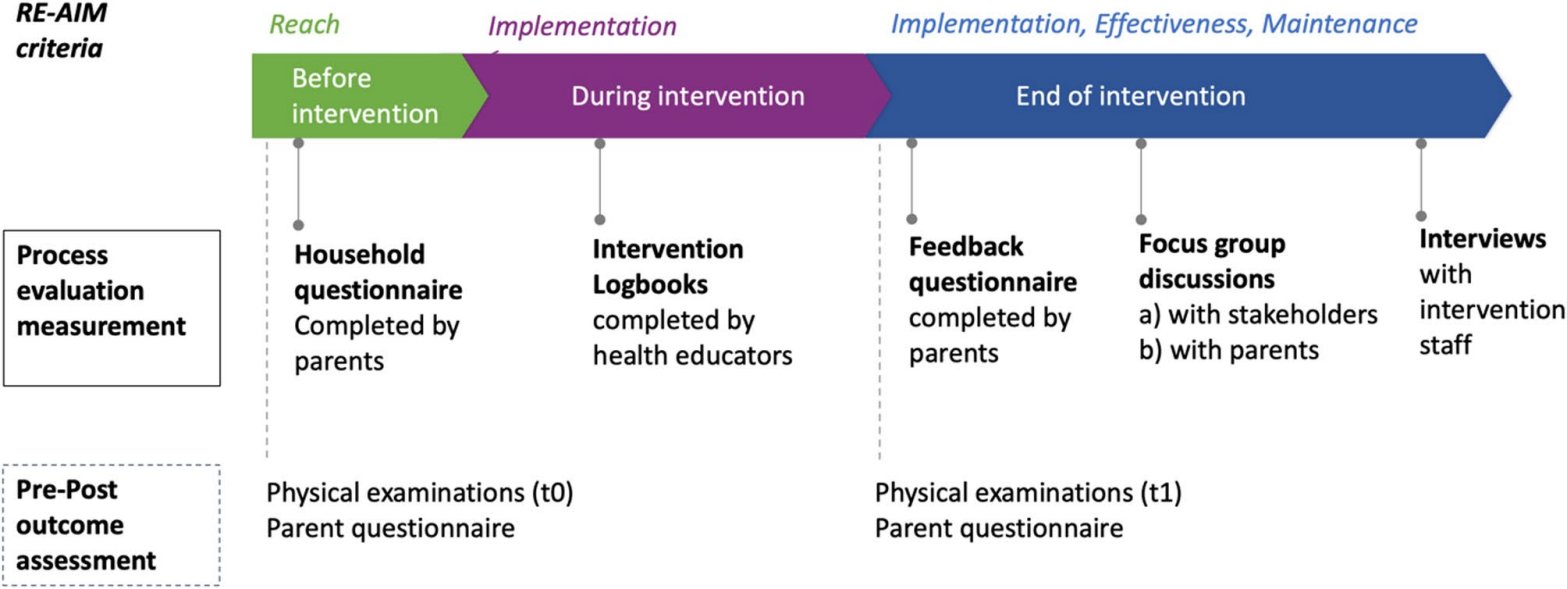
Process and pre-post outcome evaluation measurements across the study timeline

An intervention study was conducted, including a process evaluation and a pre-post outcome assessment (without control group), see Fig. 1. From the outset of the study, the research team of the Leibniz Institute for Prevention Research and Epidemiology (BIPS) liaised with an operational stakeholder (the State Association for Health and Academy for Social Medicine Lower Saxony Bremen, LVG & AFS), who was responsible for the recruitment and delivery of the intervention, and a developer of the ‘Healthy Habits, Happy Homes’ program (JH), who provided advice during the adaptation process before intervention onset. We used four of the five criteria of the RE-AIM model to evaluate this feasibility trial [30]. Adoption was left out because the intervention was implemented only in one setting and by one provider. To guide the reporting of the qualitative data of this mixed-method study, we followed the Consolidated Criteria for Reporting Qualitative Research recommendations (COREQ, Additional File 1) [31]. The research team characteristics are presented in Additional File 2. Participation in all study activities was voluntary, and all participants (including parents for their children) provided written informed consent. In addition, all children provided oral assent to the activities. The study was approved by the ethics committee of the University of Bremen, Germany (2021-18 and 2022-05).

### Participants and setting

It was planned to enroll 40 families with at least one child aged between 3 and 10 living in a socially deprived neighborhood in Bremen, Germany. Families with no child within the relevant age group were excluded. Single- and multiple-parent households were equally eligible to take part. To avoid stigmatization, no strict inclusion criteria were defined concerning indicators of social deprivation or the weight status of the children. However, we aimed to include at least 50% of families that meet one or more criteria of social deprivation: (i) migrant background, (ii) low education, (iii) social isolation, (iv) single parents, (v) financial difficulties.

Recruitment activities focused on the city district Osterholz (approx. 37 000 inhabitants), where about 75% of the children and adolescents have a history of migration and 50% of families receive social assistance. Recruitment activities took place from April 2022 to February 2023 and were supported by BIPS’s community office in Osterholz (Leibniz Living Lab, LLL). These were conducted by two health educators from LVG & AFS, who also delivered the intervention. Recruitment included both active and passive strategies [32]. Active strategies included approaching families personally (e.g. at district festivals, in parental meet-ups in local community centers), while passive strategies entailed the distribution of flyers and posters (translated into relevant languages: Turkish, Kurdish, Arabic, Russian, and English) through schools, kindergartens and other local stakeholders, as well as social media posts and ads.

### Intervention approach

The intervention is an adaptation of the ‘Healthy Habits, Happy Homes’ program, engaging the whole family system with the overall aim of fostering healthy family routines in everyday life. The core counselling technique to facilitate change is motivational interviewing (MI) [33]. MI requires that the majority of the communication lie with family members, while the role of the health educator is to ask open-ended and activating questions. Two health educators who held a professional degree in nursing and a university degree in Public Health carried out the family visits. In advance, they received a certified MI training course of nine full days delivered by the ‘GK Quest Akademie’ in Heidelberg, Germany. Additionally, they attended a one-day training session on intercultural communication and easy-to-understand language. During the visits, the health educator supports parents and their children in selecting and achieving goals for behavior change in the areas of PA, screen time, drinking water, eating fruits or vegetables, sleep, family meals or an additional topic identified by the family members. Families individually decided in which area to set a goal. Working sheets (e.g., for setting and monitoring goals) have been used to structure the visits. Overall, the intervention consists of an introductory meeting and four intervention visits (30–60 minutes each), which take place at the families’ homes or at an optional meeting space over a planned period of 4–6 months. Between the visits, families receive small gifts as a reminder and reinforcement for behavioral change (e.g., a drinking bottle to increase water intake; total value of the gifts per family: 50 euros) and tailored information material via a social media messenger. This channel (optionally email) is also intended for clarification inquiries and maintaining contact. A description of the intervention applying the TIDieR-Checklist is presented in Additional File 3.

As a prerequisite for this feasibility study, the intervention has been adapted to the German context. We used the dimensions of the Ecological Validity Model (EVM) [34] to structure the adaptation process. As a first step, intervention materials (e.g., working sheets) were provided by the program developer, and the key principles and contextual differences were discussed among partners (BIPS, LVG & AFS, University of Guelph). Subsequently, project materials were translated into German. Three focus group discussions with eligible parents (*n* = 15 mothers), and two focus group discussions with health educators working in schools in deprived areas (*n* = 14), were conducted. These sessions covered discussions about intervention goals, delivery mode, intervention materials, communication principles, and planned recruitment strategies. Participants generally considered the intervention approach and goals useful and necessary. The following components of the original intervention were kept: (i) the frequency, spacing and duration of the intervention visits, (ii) the targeted health behaviors, (iii) the use of MI as a core counseling technique, and (iv) sending intervention materials and support gifts to the families between the visits. A summary of the key adaptations that resulted from the focus group discussions and an overview about the intervention according to the EVM-dimensions can be found in Table 1.

**Table 1:** Adaptations of Healthy Habits, Happy Homes intervention for the German context

Dimensions ^a^	Components retained from original intervention Healthy Habits, Happy Homes	Changes to original intervention for the German context (GrowH! Intervention)
Goals	• Increase in physical activity, water intake, vegetable and fruit intake, sleep duration reduction of screen time • Increase in frequency of joint family meals, in time for family physical activity, healthier home environment • Increased self-efficacy for behaviour change • Families were invited to contribute further choices of targeted health behaviours by health educators in visits	-
Concepts	• Focus on whole family as a system of interconnected and interdependent individuals • Promotion of autonomous motivation and self-efficacy for behaviour change	-
Methods	•30–60 min structured monthly sessions (welcome/check in, health educators explaining behavioural areas of the intervention/discussion of progress made on the set goals, rating on health behaviours, selection of family goals) • Trained Health educators making use of the Motivational Interviewing technique • Use of working sheets to structure the visits • Families were asked to work on achieving their goals between the visits, supported by additional information material as well as behavioural supports sent by health educators	• Adding an extra session at the beginning of the intervention for strengthening trust and relation between health educators and families (*n* = 5 visits in total) • Revised design of working sheets according to suggestions of participants (35) • Revised information material (e.g. fact sheets, videos etc.) to reflect most recent German recommendations • Communication between health educators and families primarily via smart phone (e.g. WhatsApp or phone calls) • Reduction of standardization (flexibilization) of types of information materials, and behavioural supports
Language	• Intervention is delivered in English language	• Lowered reading level of intervention material in easy to understand and simple German language • Making use of translation tools (e.g. translation sticks, translation apps)
Context	• Sessions take place at the families’ homes	• Adding the option of a public but confidential and accessible meeting space offered for family visits (LLL and a neutral meeting place) • Linking families to activities and services provided in the local area (e.g. sport clubs)

^a^ Dimensions are adapted from the Ecological Validity Model (EVM) (34)

### Process and outcome evaluation

Table 2 summarizes how the process evaluation measures and outcome evaluation variables were used to evaluate the intervention according to the RE-AIM criteria.

**Table 2:** Indicators and data used for the process evaluation according to the RE-AIM criteria [30]

RE-AIM criteria	Indicators			Data collection method *		Source of data
Reach	Number of participants reached; degree of social deprivation among participants			Household questionnaire		Parents
	Recruitment channel (active vs. passive approaches)			Intervention logbook		Intervention staff
				Qual. Interviews		
				Qual. focus groups		Parents
Implementation	Fidelity of intervention (number of completed visits; location of visits; choice of topics; use of intervention material; sending of support gifts, use of MI^a^)			Intervention logbook		Intervention staff
	Acceptability of intervention (number of visits completed, potential recommendation of the program to others, satisfaction with the program)			Feedback questionnaire		Parents
				Qual. focus groups		Parents
				Qual. interviews		Intervention staff
Effectiveness	Perceived effects (perceived changes in family life)			Feedback questionnaire		Parents
				Qual. focus groups		
				Qual. interviews		Intervention staff
	Acceptability of outcome evaluation (number of families participating; perception of intervention staff)			Pre-post measurement		Parents, child
				Qual. interviews		Intervention staff
	Changes in measures of healthy weight development and behavior change			Pre-post measurement		Parents, child
		(z)BMI	weight, kg		Electronic scale^b^	
			Height, cm		Stadiometer^c^	
		PA	Accelerometers, wrist-worn (7 consec. days, 24 h/day)		GENEActiv device ^d^ (36)	
			sports club membership, yes/no		Parent questionnaire	
		Dietary habits	frequency of fruit and vegetable consumption, sugary soft drink consumption; sweet/salty snacking		Parent questionnaire (items based on WHO Childhood Obesity Surveillance Initiative (COSI) study(37))	
		Sleep duration	bed and wake-up times weekdays and weekends/vacations			
		Screen-time	hours/day spent in front of a screen (tablet, computer, smartphone, tv, e-games)			
		Behavioural problems	Total difficulties (emotional symptoms, conduct problems, hyperactivity/inattention, peer problem)		Parent questionnaire (items based on Strengths and Difficulties Questionnaire (SDQ), (38, 39))	
Maintenance	Requirements for long-term implementation (reaching the target group in the long-term, critical aspects of program delivery)			Qual. focus groups		Locally operating stakeholders
				Qual. interview		Intervention staff

* = Type of data, that is not further specified is of a quantitative nature; *BMI* body mass index, *MI* motivational interviewing; *PA* physical activity

^a^ Assessment of MI-adherence was based on subjective ratings provided by health educators (percentage to which families took the lead in a solution-focused dialogue, >50% = high)

^b^ Tanita Europe GmbH, Sindelfingen, Germany

^c^ Calibrated, Seca, Birmingham, United Kingdom

^d^ Activinsights Ltd, Kimbolton, United Kingdom

The process evaluation included an interview-led household questionnaire completed at intervention intake, covering basic sociodemographic characteristics of the families (see Additional File 4) to assess the reach of the intervention. Furthermore, the health educators completed an intervention logbook after each visit to assess the fidelity of the implementation. At the end of the intervention, parents completed a short feedback questionnaire to assess the acceptability of the implementation. Additionally, qualitative interviews with the two health educators and the project coordinator from LVG & AFS as well as two focus group discussions with participating parents (*n* = 10) were conducted to further reflect on the implementation process qualitatively. To assess the maintenance of the intervention, a focus group with locally operating stakeholders (*n* = 9) from different sectors including school and kindergarten, social work in the community, pediatric care, sports was conducted (see Additional File 5 for the topic guides of qualitative activities).

Before the start of the intervention, the families were invited to take part in a pre- and post-outcome evaluation. To test its acceptability, participation in the outcome evaluation was optional for families. They were offered 30 euros in reimbursement and individual feedback regarding the change over time in the outcome variables after participating in both measurement points. The assessments were conducted at the family’s home or at the LLL, and were carried out by trained field staff. The pre-post measurements assessed the weight status of parents and children and the dietary habits, PA, sleep duration, screen time and behavioral problems of the children. See Additional File 6 for the parent questionnaire used for the pre-post measurement.

### Analysis

#### Qualitative Analysis

Transcripts of all focus groups and interviews were reviewed and compared to the original audio recordings for quality assurance (mean interview duration was 55 min, mean focus group duration was 70 min). All transcripts were coded in MAXQDA (version 2020) by using thematic analyses [40]. A hybrid inductive-deductive approach was applied to identify patterns in the data. Initially, all transcripts were coded inductively by MLS and a codebook was developed for all study activities. To obtain different perspectives on the coding scheme, a second research assistant independently coded the transcripts to achieve consensus through discussion. After the first round of coding, a second round was carried out by MLS to refine the codes. Finally, the codes were then collated into overarching and sub-themes and classified under the criteria of the RE-AIM model (see Additional File 7 for an example of the coding frame). All quotes provided in the results section were translated by MLS.

#### Quantitative Analysis

Statistical analyses were performed using SAS Version 9.4 (SAS Institute Inc, Cary, North Carolina, USA). Information on financial difficulties (finding it (very) difficult on present income vs. coping or living comfortably on present income), parental education (less than 12 years of education and no professional degree vs. higher degree), family status (single parent vs. partnered), migration status (parent born abroad vs. born in Germany), and social isolation (only one person to rely on or less vs. 2 or more persons to rely on) from the household questionnaire were dichotomized to assess the degree of social deprivation.

Height and weight measurements were used to calculate parents’ and children’s BMI. For children the values were transformed into z-scores, and the cut-offs from the International Obesity Task Force were used to define overweight and obesity [41]. The raw accelerometer data were analyzed in R (version 4.3.2) with the package GGIR (version 3.0–0) [36] using the Phillips et al. [42] cut-points for the non-dominant wrist to estimate the weighted average minutes of moderate-to-vigorous PA (MVPA) per day, whereby weekend days were weighted 2/5 relative to the contribution of weekdays (at least 3 valid weekdays [43] and at least 1 weekend day [44], minimum 16 h wear time). Furthermore, it was assessed whether the children met the recommendation of 60 min or more MVPA per day [45, 46]. Parentally reported bed and wake-up times were used to calculate a weighted average over weekdays (weight 5/7) and weekends (weight 2/7) and it was assessed whether children met the age-specific sleep recommendation (3 to 5 years: 10–13 h; 6 to 12 years: 9–12 h [47]). For ease of interpretation, food frequencies were dichotomized to approximate food intake recommendations. For healthy foods (fruits and vegetables), the cut-off was at least once a day vs. less. For ‘sometimes’ foods, the cut-offs were once a week (sugary soft drinks) and 1 to 3 days per week or less (salty snacks, sweet snacks) vs. >1 per week and >3 days per week, respectively. Information on screen time was categorized according to age-specific recommendations (3 to 5 years: 30 minutes per day or less vs. >30 minutes per day; 6 to 10 years: up to 2 hours per day or less vs. >2 hours per day). To assess behavioral problems, the SDQ total difficulties score was calculated using the 4-band categorization (scores 0–13 = close to average, 14–16 = slightly raised, 17–19 = high, 20–40 = very high).

Change over time was only analyzed descriptively due to the lack of statistical power. Pairwise deletion was used to handle missing values.

## Results

### Reach

In total, 23 families (23 caregivers and 33 children) participated in the study. In all cases, mothers registered for the study and filled out the household questionnaire. For demographic characteristics, including indicators of social deprivation, see Table 3. At least one indicator of social deprivation was met by 87% of the participating families. More than 80% of mothers were born abroad, 39% faced financial difficulties, about 30% were single parents and socially isolated, and 17% had a low educational degree. Most of the families had more than one child (mean = 2.4) and 67% of the children within the study were girls.

Active approaches were more effective in recruiting families than passive approaches. A total of 10 out of 23 families were recruited by face-to-face contacts at local community centers or parental meet-ups, while 5 of 23 were recruited indirectly via partners in the local network (e.g., social service providers and kindergartens). Direct contact with families was of particular importance in the recruitment phase because it offered opportunities to create trust, left room for explaining the intervention in detail, and overcame language barriers. Word-of-mouth also proved to be an important recruitment strategy, resulting in 5 of 23 families being recruited by participating families. A participating mother noted:

*" I always think with the flyer you do not reach so many people… Flyers are not important for people*,* word-of-mouth is. I met J. (name of health educator)*,* and the personal connection was already really much better*,* and then J. came to my German course*,* and then friends were interested.”* [Participating mother, focus group discussion with parents].

Only 2 families were recruited via flyers and 1 family via social media channels. From the health educators’ perspective, it was challenging to convey the intervention’s elements, processes and benefits in an understandable but concise format on flyers and social media posts. At the same time, passive approaches required a certain degree of agency and motivation among the families to initiate contact with the health educators. In addition, a participating mother mentioned in the focus group discussion that some deprived families were suspicious about the home visiting approach due to negative previous experiences with state authorities (e.g., child protection services).

**Table 3:** Demographic characteristics of participating families and children in the GrowH! study

Demographic Variable			*n* (%)	Mean (SD)
Family characteristics (*n* = 23)				
	Caregiver			
		Relation to child, mother	23 (100)	
		Age (years)		37.4 (5.5)
	Indicators of social deprivation			
		Single parent	7 (30.4)	
		Migration status (mother born abroad)	19 (82.6)	
		Financial difficulties	9 (39.1)	
		Low education	4 (17.4)	
		Social isolation	7 (30.4)	
	Household characteristics			
		Number of children living at home		2.4 (1.1)
Child characteristics (*n* = 33)				
		Sex, female	22 (66.7)	
		Age (years)		6.0 (1.8)

### Implementation

#### Fidelity

The intervention (4 visits) took place over an average duration of about 5 months (range 3–9 months), and 83% of the families completed all of the visits. 50% of all health educator visits took place at the families’ homes, 35% at the LLL and 20% at alternative locations chosen by the families (most often the library or playground, rarely online). The health educator visits lasted on average 55 min (range: 16–105 min). In all cases, the mother was present, in three families, the father was also present, and most frequently, 1 to 3 children were present during the visits. The cooperation with the whole family (involving parents and children) was rated by the health educators in 55% of the visits as “good” to “very good” but in 33% as “not good at all”. In all but one of the families, the intervention was conducted in the German language (*n* = 1, English). The health educators rated the quality of communication and understanding with the families in 79% of the visits as “very good” or “good”. In about 80% of the visits, the families contributed more than 50% of the conversation share. For about one-third of the visits, translation tools were used to overcome language barriers; most often, family members assisted in translating content, followed by translation apps on smartphones. Between the visits, health educators stayed in contact with all families via WhatsApp and, in exceptional cases, also via email (6%). The health behavior goal most frequently selected by families was water intake (50% of visits), followed by screen time (46%), fruit and vegetable intake (35%), and sleep duration (31%). Less frequently, families focused on PA (17%) and family meals (13%). In 16% of the health educator visits, families set additional selected goals, specifically identified by family members, such as reducing sweet snacking. On average, families chose 3 goals (range: 1–7) across all visits. Following the visits, 93% of the cases received behavioral support gifts by mail and all families were provided with tailored information materials.

One major adaptation during the implementation was that in one case the health educator conducted group sessions with three mothers and their children instead of individual meetings. This was initiated by the mothers who knew each other beforehand and who perceived joint participation as a way to increase trust and motivation.

#### Acceptability

Only 4 families left the study prematurely, suggesting that the intervention’s implementation and procedures were acceptable to participants. Fourteen families filled out a feedback questionnaire, of which 13 reported that they would recommend the intervention to a friend. Additionally, on a scale from 1 to 5, they rated their satisfaction with the intervention on average at 4.9 (5 = very satisfied). When asked about their satisfaction with the intervention, mothers in the focus group discussion positively emphasized as facilitators the focus on small-step goals, the regularity of the visits, and the flexibility regarding the scheduling of visits. At the same time, this flexible scheduling of visits was perceived as a barrier by the health educators. Especially the frequent appointment cancellations (average rescheduled visits per family = 4), also required corresponding resources from the health educator’s perspective:

*“On the other hand*,* it was also super stressful. With the planning*,* the appointments*,* the cancellations… Yes*,* these last-minute cancellations*,* things like that.”* [Intervention staff, interview].

Even though MI was rated positively during the intervention delivery process, retrospectively language barriers were perceived by the health educators as barrier in implementing the MI technique as intended. The use of translation tools did not fully overcome these challenges and therefore could not contribute to a seamless application of the MI method.

### Effectiveness

#### Perceived effects

Thirteen of 14 families reported that they had learned something new by participating in the intervention (Mean = 4.3; 5 = very much new). However, one family reported that they have learned nothing new, and two families answered that nothing has changed in their family life as a result of participating in the intervention. Mothers participating in the focus groups agreed that they still benefit from the intervention in their daily lives. One mother, who was focusing on the reduction of her son’s screen time and the increase of PA, described personal benefits as follows:

*“So*,* with us*,* it’s like this*,* we were always into screen time and physical activity*,* and that has helped. He knows it*,* we’ve always set time limits: You can have two*,* three hours today. And it’s still the same today. We also go for more walks and walk more now. He even asks on his own: When are we going for a walk? When are we going to the playground?”* [Participating mother, focus group discussion with parents].

This was also backed by the perception of the health educators who stressed that the degree to how much the families benefited varied across the families.

*“The families have definitely all benefited*,* but in an incredibly varied range. So*,* from a very*,* very minimal benefit to ‘I have truly made a difference in my family’s habits.”* [Intervention staff, interview].

#### Acceptability of the outcome evaluation

Of the 23 families, 21 participated in the outcome evaluation and 18 completed both measurement points, which indicates high acceptability of the outcome evaluation among the families. Both the financial reimbursement and the interest in feedback from measurements motivated the families according to the perception of the health educators and the field staff. Nevertheless, the health educators experienced that the outcome evaluation procedures complicated explaining the intervention clearly to families, possibly resulting in misconceptions about the intervention among families who chose not to participate. The health educators also perceived that it was difficult for the families to understand the optional nature of the outcome evaluation due to language barriers.

#### Change in outcome variables over time

A total of 18 mothers and 28 children provided data for both measurement points. The descriptive comparison of the pre- and post-measurements show that mothers’ and children’s BMI did not change over time (Table 4). Positive trends were observable for PA and screen time. MVPA of the children was already on a high level at baseline and was even higher after the intervention. Complementary to this, more children were members of a sports club after the intervention. The data indicate that screen time was reduced both on weekdays and on weekends. No change was observable for sleep duration and no positive trend was observable in any of the outcome variables concerning dietary habits. The number of children with increased values in the total difficulties scores slightly decreased over the course of the intervention.

**Table 4:** Pre- and post-measurement of the outcome variables

Outcome Variable		Pre		Post	
	*n*	Frequency	%/M (SD)	Frequency	%/M (SD)
Mothers’ BMI	15		27.3 (5.6)		27.2 (5.4)
Overweight/obese	15	9	60%	9	60%
Children’s zBMI	27		0.2 (1.4)		0.3 (1.3)
Overweight/obese	27	5	18.5%	5	18.5%
Physical activity					
MVPA (weighted average minutes per day)	16		78.0 (19.6)		86.3 (23.9)
MVPA recommendation met	16	14	87.5%	14	87.5%
Sports club membership	28	6	21.4%	10	35.7%
Screen-time					
Recommendation met (weekdays)	27	16	59.3%	21	77.8%
Recommendation met (weekends)	26	10	38.5%	12	46.2%
Sleep					
Sleep duration (weighted average hours per night)	28		10.9 (0.7)		10.8 (0.7)
Sleep recommendation met	28	28	100%	28	100%
Dietary habits					
Fresh fruits, at least once a day	28	22	78.6%	21	75.0%
Vegetables, at least once a day	28	16	57.1%	14	50.0%
Sugary soft drinks, less than once a week	28	24	85.7%	18	64.3%
Salty snacks, sometimes (1–3 days per week or less)	28	27	96.4%	24	85.7%
Sweet snacks, sometimes (1–3 days per week or less)	28	20	71.4%	17	60.7%
Behavioural problems (total difficulties score, SDQ)	28				
Average		22	78.6%	25	89.3%
Slightly raised		3	10.7%	2	7.1%
High		2	7.1%	1	3.6%
Very high		1	3.6%	-	-

*BMI* body mass index, *MVPA* moderate-to-vigorous physical activity, *SDQ* Strengths and Difficulties Questionnaire

### Maintenance

Locally operating stakeholders including pediatricians, a representative of the federal sports association, a social worker from a primary school, and coordinators of kindergartens and community centers discussed perspectives on the long-term implementation of the intervention in the city district Osterholz. Overall, the stakeholders saw a demand for a varied range of obesity prevention initiatives, where the GrowH! intervention, particularly if it focuses on families with young children (kindergarten age or younger), could play a meaningful role. However, the integration of the intervention within institutions like kindergartens or community centers that provide families with a familiar environment and a trusted contact person was seen as a crucial requirement for intervention sustainability. With regard to a successful recruitment strategy, a kindergarten coordinator shared from her professional experience:

*“When we try to refer them [the families] elsewhere*,* this is often a great barrier. So*,* if you connect such a program to a kindergarten or a family center*,* I believe the success is greater because I simply think they need a trusting environment that they know*,* not having to meet someone new again*,* not having to go to someone else*,* and not knowing what to expect.”* [Coordinator of a local kindergarten, focus group discussion with stakeholders].

Regarding intervention delivery, stakeholders viewed health educator visits at the families’ homes as an important option but emphasized the need for a sensitive, non-intrusive approach. Moreover, the discussants proposed that adding more practical elements that go beyond counselling (e.g., cooking together) to the visits could enhance the attractiveness of the intervention. Further considerations for the future implementation were expressed by intervention staff in an interview after the intervention. To enhance its long-term effectiveness, the strategic referral of families to subsequent health promotion programs was suggested. For this purpose, an integrated community-wide approach that strategically coordinates the existing opportunities for health promotion and prevention was recommended.

## Discussion

In this study, we assessed the feasibility of implementing a family-based intervention, developed in the US/Canadian context, in a socially deprived and ethnically diverse neighborhood in Bremen, Germany. Our results show that, although the overall recruitment target of 40 families was not reached, the targeted percentage of families that reported at least one indicator of social deprivation was exceeded. In reaching these families, active recruitment strategies prove more successful than passive strategies. The fidelity and acceptability of the intervention were generally rated high, but the use of MI was somewhat compromised due to language barriers. Participation in the intervention was perceived as beneficial for the families, although it varied across the families. Analyzing the change over time in relevant outcome variables descriptively showed positive trends in PA, screen time, and behavioral problems, but none in BMI, dietary habits or sleep duration. For long-term implementation, stakeholders suggested an integration of the intervention into available structures, e.g., kindergartens, to increase reach and effectiveness.

Recruiting families in disadvantaged neighborhoods for an obesity prevention program that is delivered in a research context is a difficult task. Unlike the testing of the program in the Canadian context [24], recruitment through websites and social media channels was not very effective. As demonstrated in previous research, our findings indicated that active recruitment strategies reach socially deprived participants for obesity prevention programs better, even though the costs are much higher than for passive recruitment strategies [32]. The importance of face-to-face meetings for an effective recruitment process was also highlighted in a feasibility trial of the program in Scotland [48]. In this context, the crucial role of trust-building when reaching and engaging with socially deprived families need to be highlighted [49, 50]. Efforts to build trust in initial encounters, as well as in transferring trust from a trusted person, e.g., the kindergarten coordinator, to the health educator, require high priority.

The implementation of the intervention proved to be feasible and acceptable, consistent with similar studies conducted in Canada and Scotland. These studies emphasized the benefits of the home visiting approach for families [24] and the importance of flexibility in scheduling visits [48]. However, the Scottish study also identified challenges, including a high administrative workload resulting from visit cancellations, and difficulties in applying MI in hectic and distracting family environments [48]. Similarly, in our study, adhering to MI principles proved difficult for health educators in certain situations. Language barriers, in particular, made it challenging to establish a fluent conversational flow, which hindered families’ ability to clearly articulate goals, reflect on them, and engage in a solution-focused dialogue. This underscores the necessity for the culturally sensitive adaptation, particularly of MI, that consider the diversity among ethnic and cultural minorities, as recommended in current research [51]. Our findings suggest that either specific training courses for intervention staff are required to implement MI in families with low language skills [52, 53], or a modification of the intervention content that offers more practical skill-building activities, e.g., cooking classes. Especially in culturally adapted interventions using MI, incorporating additional elements, such as community support events is common [51].

To achieve a longer-term implementation, this feasibility trial showed the need for more extensive adaptations of the intervention (e.g., adding practical elements to the intervention, strategic referral to other local services or offers). This highlights the need for future pilot runs of the intervention, as also explained in the ADAPT guideline [28]. Locating the intervention at an established institution or organization in the neighborhood and making it a part of a larger action plan to combat childhood obesity were additional suggestions for adaptation. While this does not necessarily imply changes to the basic structure of the intervention, it still requires re-examining interfaces and connections with kindergarten and school-based obesity prevention activities.

Overall, this feasibility trial showed promising results in terms of transferability and effectiveness of the intervention. Specifically, increasing sports club membership was recently identified as one of the most important factors for childhood obesity prevention in socially deprived families in the long run [54]. However, no positive trends were observed in the areas of sleep, screen time, and BMI, unlike in other studies [23–25].

Major limitations of this study are the small sample size and the lack of a control group, which are due to the nature of a feasibility trial. Findings about changes in the outcome variables are therefore only descriptive and not appropriate to establish the efficacy of the intervention. Although we used established questionnaires to collect information on dietary habits, sleep, and screen time, the data are still based on parent reports and are thus prone to social desirability. We used complete case analysis. We cannot rule out that those who dropped out differed in terms of behavior change from those who participated in the follow-up. It should be noted that only 61% of the families returned the feedback questionnaire. It is unclear whether those who returned it differed in baseline characteristics from those who did not complete it. In the process evaluation, MI-adherence was not objectively measured through audio recordings. Only the health educators’ subjective assessments were available.

## Conclusion

Although recruitment of families is challenging, this study demonstrated that the ‘Healthy Habits, Happy Homes’ program is acceptable to families in deprived neighborhoods in Bremen, Germany, and is potentially beneficial for them. Nevertheless, more adaptations of the intervention are needed to overcome language barriers and integrate the intervention into community-wide approaches on childhood obesity prevention.

## Supplementary Information

Supplementary Material 1.

Supplementary Material 2.

Supplementary Material 3.

Supplementary Material 4.

Supplementary Material 5.

Supplementary Material 6.

Supplementary Material 7.

## Data Availability

The datasets used and analyzed during the current study are available from the corresponding author on reasonable request.

## Abbreviations

BIPS: Leibniz Institute for Prevention Research and Epidemiology
BMI: Body mass index
COREQ: Consolidated Criteria for Reporting Qualitative Research
COSI: WHO Childhood Obesity Surveillance Initiative
GrowH! project: Growing up healthy: obesity prevention tailored to critical transition periods in the early life-course
LLL: Leibniz Living Lab
LVG & AFS: Landesvereinigung für Gesundheit & Akademie für Sozialmedizin (State Association for Health and Academy for Social Medicine Lower Saxony Bremen)
MI: Motivational interviewing
MVPA: Moderate-to-vigorous physical activity
PA: Physical activity
RCT: Randomized Controlled Trial
SDQ: Strengths and Difficulties Questionnaire
