# Supplementary material for: Implementing a family-based intervention to promote healthy family routines in deprived neighborhoods – a feasibility study from Bremen, Germany
Source: BMC Public Health. 2025 Dec 23;25:4344. doi: 10.1186/s12889-025-25532-9 (PMC12751738; doi:10.1186/s12889-025-25532-9)
Supplement: Supplementary file 1 — Supplementary Material 1. [file 12889_2025_25532_MOESM1_ESM.docx]

**Additional file 1**

**Consolidated criteria for reporting qualitative studies (COREQ): 32-item checklist (1)**

| **No. Item** | **Guide questions/description** | **Reported on Page #** | |
| --- | --- | --- | --- |
| **Domain 1: Research team and reflexivity** | | | |
| *Personal Characteristics* | | | |
| 1. Interviewer/facilitator | Which author/s conducted the interview or focus group? | Additional file 2 | |
| 2. Credentials | What were the researcher’s credentials? E.g. PhD, MD | Additional file 2 | |
| 3. Occupation | What was their occupation at the time of the study? | Additional file 2 | |
| 4. Gender | Was the researcher male or female? | Additional file 2 | |
| 5. Experience and training | What experience or training did the researcher have? | Additional file 2 | |
| *Relationship with participants* | | | |
| 6. Relationship established | Was a relationship established prior to study commencement? | Additional file 2 |  |
| 7. Participant knowledge of the interviewer | What did the participants know about the researcher? e.g. personal goals, reasons for doing the research | Additional file 2 |  |
| 8. Interviewer characteristics | What characteristics were reported about the interviewer/facilitator? e.g. bias, assumptions, reasons and interests in the research topic | Additional file 2 |  |
| **Domain 2: study design** | | |  |
| *Theoretical framework* | | |  |
| 9. Methodological orientation and Theory | What methodological orientation was stated to underpin the study? e.g. grounded theory, discourse analysis, ethnography, phenomenology, content analysis | Methods, page 11 |  |
| *Participant selection* | | |  |
| 10. Sampling | How were participants selected? e.g. purposive, convenience, consecutive, snowball | Methods, page 6 and 10 |  |
| 11. Method of approach | How were participants approached? e.g. face-to-face, telephone, mail, email | Methods, page 6 Results, page 13 |  |
| 12. Sample size | How many participants were in the study? | Methods, page 10 Results, page 12 |  |
| 13. Non-participation | How many people refused to participate or dropped out? Reasons? | Results, page 15 |  |
| *Setting* | | |  |
| 14. Setting of data collection | Where was the data collected? e.g. home, clinic, workplace | Methods, page 6 |  |
| 15. Presence of non-participants | Was anyone else present besides the participants and researchers? | N/A |  |
| 16. Description of sample | What are the important characteristics of the sample? e.g. demographic data, date | Methods, page 10,  Results, page 12 |  |
| *Data collection* | | |  |
| 17. Interview guide | Were questions, prompts, guides provided by the authors? Was it pilot tested? | Additional file 5 |  |
| 18. Repeat interviews | Were repeat interviews carried out? If yes, how many? | N/A |  |
| 19. Audio/visual recording | Did the research use audio or visual recording to collect the data? | Methods, page 11 |  |
| 20. Field notes | Were field notes made during and/or after the interview or focus group? | N/A |  |
| 21. Duration | What was the duration of the interviews or focus group? | Methods, page 11 |  |
| 22. Data saturation | Was data saturation discussed? | N/A |  |
| 23. Transcripts returned | Were transcripts returned to participants for comment and/or correction? | N/A |  |
| **Domain 3: analysis and findings** | | |  |
| *Data analysis* | | |  |
| 24. Number of data coders | How many data coders coded the data? | Methods, page 11 |  |
| 25. Description of the coding tree | Did authors provide a description of the coding tree? | Additional file 7 |  |
| 26. Derivation of themes | Were themes identified in advance or derived from the data? | Methods, pages 11 |  |
| 27. Software | What software, if applicable, was used to manage the data? | Methods, page 11 |  |
| 28. Participant checking | Did participants provide feedback on the findings? | N/A |  |
| *Reporting* | | |  |
| 29. Quotations presented | Were participant quotations presented to illustrate the themes/findings? Was each quotation identified? e.g. participant number | Results, pages 12-19 |  |
| 30. Data and findings consistent | Was there consistency between the data presented and the findings? | Results, pages 12-19 |  |
| 31. Clarity of major themes | Were major themes clearly presented in the findings? | Results, pages 12-19 |  |
| 32. Clarity of minor themes | Is there a description of diverse cases or discussion of minor themes? | Results and Discussion, pages 12-21 |  |

1. Tong A, Sainsbury P, Craig J. Consolidated criteria for reporting qualitative research (COREQ): a 32-item checklist for interviews and focus groups. International journal for quality in health care. 2007;19(6):349-57.
