# Supplementary material for: Implementing a family-based intervention to promote healthy family routines in deprived neighborhoods – a feasibility study from Bremen, Germany
Source: BMC Public Health. 2025 Dec 23;25:4344. doi: 10.1186/s12889-025-25532-9 (PMC12751738; doi:10.1186/s12889-025-25532-9)
Supplement: Supplementary file 2 — Supplementary Material 2. [file 12889_2025_25532_MOESM2_ESM.docx]

**Additional file 2: Research team and reflexivity**

**Authors:**

Authors: Marie Lisanne Schepan^1,2^*, Wolfgang Ahrens^1^, Thomas Altgeld^3^, Heide Busse^1^, Jess Haines^4^, Elisabeth Rataj^3^, Annika Swenne^1^, Maike Wolters^1^, Hajo Zeeb^1^, Tilman Brand^1^

^1^Leibniz Institute for Prevention Research and Epidemiology – BIPS, Germany

^2^ University of Bremen, Bremen, Germany

^3^ State Association for Health and Academy for Social Medicine Lower Saxony Bremen, Germany (Landesvereinigung für Gesundheit und Akademie für Sozialmedizin Niedersachsen Bremen e. V.)

^4^ University of Guelph, Canada

| **Characteristic** | **Description** |
| --- | --- |
| Credentials | WA, HZ, JH are professors. TB, HB and MW hold a doctoral degree. MLS and AS are doctoral candidates and research assistants. ER is a researcher and TA a psychologist. |
| Occupation | MLS, WA, HB, TB, AS, MW and HZ work at the Leibniz Institute for Prevention Research and Epidemiology -BIPS: WA and HZ as professors, TB, HB, MW as a senior researchers and MLS and AS as doctoral candidates and research assistants.  ER and TA work at the State Association for Health and Academy for Social Medicine Lower Saxony Bremen, Germany: TA as Chief Executive Officer and ER as project coordinator. JH works at the University of Guelph, Canada as a professor in the Department of Family Relations and Applied Nutrition. |
| Gender | MLS, ER, HB, JH, ER, AS and MW self-identified as women; WA, TA, HZ and TB self-identified as men. |
| Experience and training | The authors have a background in Epidemiological Methods and Etiological Research, Public Health, Applied Nutrition, Social Medicine and Psychology and have worked with qualitative and quantitative data. |
| Relationship between participants and  researchers | MLS, who conducted the interviews and focus groups did not know any of the participants prior to the study. |
| Participant knowledge of the interviewer | MLS conducted the interviews and focus group discussions. The participants knew that the interviewer was a researcher at the Leibniz Institute for Prevention Research and Epidemiology - BIPS. They were provided with written information about the aims of the project. |
| Interviewer characteristics | MLS is a psychologist (M.Sc.) and her research interest is child health, health inequalities and target group-specific adaptation and implementation of outreach prevention programs. |
