# Supplementary material for: Implementing a family-based intervention to promote healthy family routines in deprived neighborhoods – a feasibility study from Bremen, Germany
Source: BMC Public Health. 2025 Dec 23;25:4344. doi: 10.1186/s12889-025-25532-9 (PMC12751738; doi:10.1186/s12889-025-25532-9)
Supplement: Supplementary file 4 — Supplementary Material 4. [file 12889_2025_25532_MOESM4_ESM.pdf]

☐☐☐

Household-ID

Leibniz-Institut
für Präventionsforschung und
Epidemiologie – BIPS

GrowH!

Gesund aufwachsen
Sağlıklı bir şekilde yetişmek
Bi tenduristî mezin diben
Рости здоровым
النمو بصحة جيدة

Household Questionnaire

to be completed by one parent

Dear parents or dear legal guardians,

Thank you for participating in this study.
In this questionnaire, we would like to learn a few things about you and the people living in your household.

Please answer the questions accurately and honestly. We assure you that your information will be treated absolutely confidentially. If you do not wish to answer some questions, please cross them out completely.

Thank you for your support!

Instructions for filling out the questionnaire

The questionnaire contains questions and statements to be marked with a cross. Please give one answer, unless stated otherwise.	❍
If you are asked to write down text, please use the lines provided-	________________
For questions referring to a quantity or date, please use the boxes for filling in the required data.	☐☐
Only skip a question when the following applies:	⇨ "Please continue with question..."
If you want to amend a written answer, please cross out the written words and enter the corrected answer above the cancelled words.	12<br>~~1 1~~ years old
If you would like to correct a marked answer, please completely cross out the wrong answer and mark the desired answer.	~~☒ 1 Breakfast~~<br>☒ 2 Lunch

Date of completion: ☐☐ ☐☐ ☐☐☐☐
day month year

GENERAL INFORMATION ABOUT THE HOUSEHOLD

First, we would like to know something about yourself, which means the person who is filling out this questionnaire.

1. What is your date of birth?

☐☐ ☐☐ ☐☐☐☐
day month year

2. What is your sex? *Please mark only one answer.*

Male ○ 1

Female ○ 2

Diverse ○ 3

3. What is your relationship to the child?
Please mark only one answer.

I am the mother ○ 1

I am the father ○ 2

Other (e.g. grandmother, uncle, unrelated person) ○ 3

→ I am: ____________________

4. What is your relationship status?

Single ○ 1

Partnered, living together ○ 2

Partnered, not living together ○ 3

Other, ○ 4

→ please specify: ____________________

5. **For the place where your child lives all or most of the time (> 50%), please indicate the number of people, in each box, who live there.**
Also count half siblings/step siblings.

Number of people
(if none, insert "0")

Children .... ☐

Mother .... ☐

Father .... ☐

Stepmother or girlfriend / partner .... ☐

Stepfather or boyfriend / partner .... ☐

Grandfathers .... ☐

Grandmothers .... ☐

Someone else, ... ☐

⇨ and that is: ______________________________

6. **How many children live permanently in the household where your child usually lives?**

Number of children ☐☐

How old are the children living in your household?

Child 1	☐☐	Child 6	☐☐
Child 2	☐☐	Child 7	☐☐
Child 3	☐☐	Child 8	☐☐
Child 4	☐☐	Child 9	☐☐
Child 5	☐☐	Child 10	☐☐

7. What is the highest educational degree you or your spouse or partner has completed?
Please select only one answer for each of you.

	You	Spouse / partner
No school degree (yet)	○ 1	○ 1
Graduation from a lower secondary school or intermediate secondary school (after grade 9 or 10)	○ 2	○ 2
Graduation from upper secondary school or specialized upper secondary school (after grade 12 or 13)	○ 3	○ 3
Other school degree, ⇨ please specify:	○ 4 ____________	○ 4 ____________

8. What is the highest professional degree you or your spouse or partner has completed?
Please select only one answer for each of you.

	You	Spouse / partner
Apprenticeship (vocational-in-company training)	○ 1	○ 1
Vocational school, business school (vocational-school education)	○ 2	○ 2
Professional school (e.g. master technician school, vocational or technical academy)	○ 3	○ 3
Bachelor's degree from a university or college	○ 4	○ 4
Diploma or master's degree from a university or college	○ 5	○ 5
No professional degree (yet)	○ 6	○ 6
Other degree, ⇨ please specify:	○ 7 ____________	○ 7 ____________

9. What is the main occupation of you and your spouse/partner over the last 6 months?
Please select only one answer for each of you.

	You	Spouse or partner
Domestic housework/homemaker (full-time)	◯ 1	◯ 1
Full time paid work	◯ 2	◯ 2
Work part-time	◯ 3	◯ 3
Unemployed/looking for a job	◯ 4	◯ 4
In education (full-time)	◯ 5	◯ 5
Permanently sick or disabled	◯ 6	◯ 6
Something else, ⇨ please specify:	◯ 7 ________	◯ 7 ________

10. Which of the descriptions on this card comes closest to how you feel about your household's income nowadays? *Please tick one box.*

Living comfortably on present income.	◯ 1
Coping on present income.	◯ 2
Finding it difficult on present income.	◯ 3
Finding it very difficult on present income.	◯ 4

11. Was your child born in Germany?

Yes ……… ◯ 1

No ………. ◯ 2

⇨ born in: ________________________________

12. Was the child's mother born in Germany?

Yes ……… ◯ 1

No ………. ◯ 2

⇨ born in: ________________________________

13. Was the child's father born in Germany?

Yes ……… ◯ 1

No ………. ◯ 2

⇨ born in: ________________________________

14. In what language(s) do you usually speak with your child at home?

German	◯ 1
Other language, ⇨ please specify:	◯ 2 ______________________

15. If you were born in a country other than Germany: How would you rate your German? *Please mark only one answer.*

Very good	◯ 1
Good	◯ 2
Fair	◯ 3
Poor	◯ 4
Very poor	◯ 5

16. How many persons - including your family - do you know that you can definitely rely on if you need help? *Please mark only one answer.*

Nobody	◯ 1
1 person	◯ 2
2 to 3 persons	◯ 3
More than 3 persons	◯ 4

Thank you for answering these questions!

Please check once more that you have answered in full.

Field work notes:

____

____
