# Supplementary material for: Implementing a family-based intervention to promote healthy family routines in deprived neighborhoods – a feasibility study from Bremen, Germany
Source: BMC Public Health. 2025 Dec 23;25:4344. doi: 10.1186/s12889-025-25532-9 (PMC12751738; doi:10.1186/s12889-025-25532-9)
Supplement: Supplementary file 5 — Supplementary Material 5. [file 12889_2025_25532_MOESM5_ESM.docx]

**Additional file 5: Topic guides for qualitative study activities**

1. **Topic guide I: Focus group discussion with stakeholders**

*-* *Original, German version -*

**Leitfaden für Fokusgruppendiskussionen mit Kooperationspartner*innen zur Evaluation des Gesundheitsförderungsprogramms GrowH!**

Vorstellung des Programms und Reflexion der Umsetzungserfahrungen:

- Input durch die moderierende Person: Vorstellung des Hausbesuchsprogramms und erster Umsetzungserfahrungen
- Falls Sie direkte Berührungspunkte mit dem Programm hatten:
  - Welche Erfahrungen haben Sie mit dem familienbasierten Programm gemacht?
  - Gibt es Ihrer Meinung nach Aspekte, die die Programmdurchführung erschwert oder behindert haben?
  - Welche Aspekte haben Ihrer Meinung nach zum Gelingen des Programms beigetragen?

Langfristige Implementierung des Programms und Systemperspektive:

- Es gibt viele Angebote und Maßnahmen um das gesunde Aufwachsen zu fördern: Welche Rolle kann das GrowH!-Programm dabei spielen?
- Was denken Sie: Was müssen Entscheidungsträger wissen, um das GrowH!-Programm in ihrem Stadtteil oder ihrer Kommune in die Praxis langfristig umzusetzen?
- Wenn Sie auf das Thema „Gesund aufwachsen“ aus einer übergreifenden Perspektive blicken: Was sind aus Ihrer Sicht die entscheidenden Stellschrauben, um Kindergesundheit zu verbessern?

*- Translated, English version -*

**Topic Guide for Focus Group Discussions with Collaborators/ Stakeholders for the Evaluation of the GrowH!-program**

Introduction of the Program and Reflection on Implementation Experiences:

- Input by the moderating person: Introduction of the family-based program and initial implementation experiences
- If you have had direct contact with the program:
  - What experiences have you had with the program?
  - In your opinion, are there aspects that have made program implementation difficult or hindered it?
  - What aspects do you believe have contributed to the success of the program?

Long-term Implementation of the Program and Systems Perspective:

- There are many offers and measures to promote healthy growth: What role can the GrowH! program play in this?
- What do you think: What do decision-makers need to know to implement the GrowH! program in their neighborhood or municipality in the long term?
- When you look at the topic of "Growing up healthy" from a comprehensive perspective: In your opinion, what are crucial factors to improve child health?

1. **Interview guide I: Interview with the project coordinator of the operational stakeholder (LVG&AFS)**

*- Original, German version-*

**Leitfaden für das Interview mit der Projektkoordinatorin des Praxispartners LVG&AFS zur Evaluation des Gesundheitsförderungsprogramms GrowH!**

Programmtreue und Anpassung des Programms:

- Wenn das Programm weitergeführt oder an anderen Orten umgesetzt werden würde: Welche Änderungen in der Programmkonzeption würdest du empfehlen?

Zugang und Akzeptanz:

- Wenn das Programm weitergeführt oder an anderen Orten umgesetzt werden würde: Wie lassen sich Zugang und Akzeptanz des Programms bei den Familien sicherstellen?
- Für welche Familien eignet sich das Programm aus deiner Sicht am besten?

Umsetzungssetting und Personal:

- In welchem Setting (z.B. Schule, Kita, offene Quartiersarbeit) und in welchem Format (Einzelberatung oder Gruppe; zu Hause oder an einem anderen Ort) lässt sich das Programm am besten umsetzen?
- Welche Kompetenzen und Qualifikationen des Personals sind wichtig, um das Programm mit den Familien umzusetzen?
- Welche Berufsgruppen kommen für die Umsetzung infrage?
- In welchen Bereichen müssen die Fachkräfte, die das Programm umsetzen, geschult werden?
- Welche Hintergrundbetreuung der Fachkräfte ist erforderlich?
- Welche Infrastruktur muss vorhanden sein, um das Programm es auf weitere Standorte auszuweiten?

Nachhaltigkeit:

- Welche Strukturen und Prozesse braucht es, damit das Programm langfristig und qualitätsgesichert an vielen Standorten durchgeführt werden kann?

Abschluss:

- Wir sind nun am Ende des Interviews angelangt. Gibt es noch etwas, das bisher im Interview noch nicht zur Sprache gekommen ist, was dir aber wichtig ist?

*- Translated, English version -*

**Topic Guide for the Interview with the Coordinator of the Operational Stakeholder (LVG&AFS)**

Program Fidelity and Program Adaptation:

- If the program were to be continued or implemented in other locations: What changes in the program concept would you recommend?

Access and Acceptance:

- If the program were to be continued or implemented in other locations: How can access to and acceptance of the program by families be ensured?
- In your opinion, which families do you believe the program is best suited for?

Implementation Setting and Personnel:

- In which setting (e.g., school, daycare, open neighborhood work) and in what format (individual counseling or group; at home or at another location) can the program be best implemented?
- What competencies and qualifications of staff are important to implement the program with families?
- Which professional groups are suitable/necessary for program implementation?
- In which areas do the professionals/ program implementers need to be trained?
- What kind of background support/ supervision of professionals will be required?
- What infrastructure must be in place to expand the program to additional locations?

Sustainability:

- What structures and processes are needed to ensure that the program can be conducted in the long term and with quality assurance at many locations

Conclusion:

- We have now reached the end of the interview. Is there anything else that has not been discussed in the interview yet but is important to you?

**3) Interview guide II: Interview with health educators of the operational stakeholder (LVG&AFS)**

*- Original, German version* -

**Leitfaden für das Interview mit Gesundheitsfachkräften des Praxispartners LVG&AFS zur Evaluation des Gesundheitsförderungsprogramms GrowH!**

Allgemeine Erfahrung und Zufriedenheit mit der Programmdurchführung:

- Wie war es für dich als Gesundheitsfachkraft das Programm GrowH! durchzuführen?

Rekrutierung von Familien:

- Welche Rekrutierungswege waren erfolgreich? Was könnten Gründe dafür sein?
- Welche Rekrutierungswege waren ineffizient? Was könnten Gründe dafür sein?
- Wenn die Studie jetzt noch einmal durchgeführt werden würde, was würdest du bei der Rekrutierung der Familien anders machen?
- Wie müsste das Programm deiner Meinung nach konzipiert sein oder angeworben werden, damit es von der Zielgruppe auch nachgefragt wird?
- Wurden deiner Einschätzung nach diejenigen Familien erreicht, die auch den Bedarf an solch einem Gesundheitsförderungsprogramm haben?
  - Falls nein: Welche Familien wurden deiner Meinung nach erreicht?

Programmimplementierung:

- Wie war es für dich, das Programm an einem flexiblen Ort (zu Hause, LLL, Anderswo) durchzuführen?
  - Was hat sich bewährt?
  - Wie war es für dich die Familien zu Hause zu besuchen?
- Wie würdest du die Kommunikation mit den Familien während der Treffen aber auch zwischendurch bewerten?
- Inwieweit hat die Fortbildung in motivierender Gesprächsführung dir bei der Beratung der Familien geholfen?
- Gibt es Dinge, die bei der Durchführung vom Programm besonders gut geklappt haben oder besonders hilfreich waren?
- Gibt es Dinge, die die Programmdurchführung erschwert haben?
  - Falls ja: Welche und woran könnte das gelegen haben?
- Welche Probleme oder Herausforderungen sind während der Programmdurchführung aufgetreten?
- Kam es während der Durchführung zu einer Anpassung des Programms bzw. wurde das Programm teilweise anders durchgeführt als geplant?
  - Falls ja: warum?
- Mit welchen anderen Angeboten oder Akteuren hast du zusammengearbeitet?
  - Mit welchen anderen Angeboten oder Akteuren sollte bei einer langfristigen Umsetzung intensiver zusammengearbeitet werden?
- Wenn das Programm weiterhin angeboten werden würde, gäbe es Dinge, die verändert werden sollten?
  - Falls ja: Welche Aspekte sollten verändert werden und warum?

Subjektive Wirksamkeit:

- Hat das Programm deiner Meinung nach gesunde Routinen bzw. das Gesundheitsverhalten der Familien positiv beeinflusst?
  - Falls nein: Was könnten Gründe dafür sein?
  - Falls ja: Wird das Programm deiner Meinung nach auch längerfristige positive Auswirkungen auf das Gesundheitsverhalten der Familien haben?

Abschluss:

- Wir sind nun am Ende des Interviews angelangt. Gibt es noch etwas, das bisher im Interview noch nicht zur Sprache gekommen ist, was dir aber wichtig ist?

*- Translated, English version -*

**Topic Guide for the Interview with Health Educators of the Operational Stakeholder (LVG&AFS)**

General Experience and Satisfaction with Program Implementation:

- How was your experience as a healthcare professional to implement the GrowH! program?

Recruitment of Families:

- Which recruitment methods were successful? What could be the reasons for this?
- Which recruitment methods were inefficient? What could be the reasons for this?
- If the study were to be conducted again, what would you do differently in recruiting families?
- In your opinion, how should the program be designed or promoted to be demanded by the target audience?
- Do you believe that the families in need of such a health promotion program were reached?
  - If not: In your opinion, which families were reached?

Program Implementation:

- How was it for you to implement the program at different locations (at home, at other locations)?
- What worked well?
- How was it for you to visit families at their homes?
- How would you evaluate communication with families during meetings and in between?
- To what extent did training in motivational interviewing help you in counseling families?
- Are there things that worked particularly well or were particularly helpful during program implementation?
- Were there any difficulties in program implementation?
  - If yes: What were they and why?
- What problems or challenges arose during program implementation?
- Was there any adaptation of the program during implementation or was the program carried out differently than planned?
  - If yes: Why?
- With which other services or actors did you collaborate?
- With which other services or actors should there be more intensive collaboration in long-term implementation?
- If the program were to continue to be offered, are there things that should be changed?
  - If yes: What aspects should be changed and why?

Subjective Effectiveness:

- Do you believe that the program positively influenced healthy routines or the health behavior of families?
  - If no: What could be the reasons for this?
  - If yes: Do you believe the program will have longer-term positive effects on the health behavior of families?

Conclusion:

- We have now reached the end of the interview. Is there anything else that has not been discussed in the interview yet but is important to you?
