# Supplementary material for: Implementing a family-based intervention to promote healthy family routines in deprived neighborhoods – a feasibility study from Bremen, Germany
Source: BMC Public Health. 2025 Dec 23;25:4344. doi: 10.1186/s12889-025-25532-9 (PMC12751738; doi:10.1186/s12889-025-25532-9)
Supplement: Supplementary file 6 — Supplementary Material 6. [file 12889_2025_25532_MOESM6_ESM.pdf]

Participant ID

Household ID

Leibniz-Institut
für Präventionsforschung und
Epidemiologie – BIPS

GrowH!

Gesund aufwachsen
Sağlıklı bir şekilde yetişmek
Bi tenduristî mezin diben
Рости здоровым
النمو بصحة جيدة

Parent Questionnaire (T0)

to be filled in about your child of primary school age (1st/2nd grade)

Dear parents or dear legal guardians,

Thank you for participating in this study!

In this questionnaire, we would like to ask you about your child's health and health related behavior, such as diet and physical activity. Please answer the questions accurately and honestly. We assure you that your information will be treated with absolute confidentiality.

Since not all children live with their biological parents, the questionnaire can also be completed by other persons with whom the child lives. When the questionnaire refers to "your child," it always means the participating **child of primary school age (1st/2nd grade)**, regardless of the exact relationship to the child.

Participation in the questionnaire is voluntary. If you do not wish to answer some questions, please cross them out completely.

Thank you for your support!

Instructions for filling out the questionnaire

The questionnaire contains questions and statements to be marked with a cross. Please give one answer, unless stated otherwise.	❍
If you are asked to write down an answer as text, please use the lines provided.	________________
For questions referring to a quantity or date, please use the boxes for filling in the required data.	☐☐
Skip a question only when the following applies:	⇨ "Please continue with question..."
If you want to amend a written answer, please cross out the written words and enter the corrected answer above the cancelled words.	12<br>~~1 1~~ years old
If you would like to correct a marked answer, please completely cross out the wrong answer and mark the desired answer.	☒ 1 ~~Breakfast~~<br>☒ 2 Lunch

Date of completion: ☐☐ ☐☐ ☐☐☐☐

day month year

GENERAL INFORMATION ABOUT YOUR CHILD

What is the date of birth of your child? ☐☐ ☐☐ ☐☐☐☐

Day Month Year

What sex is your child?

Male ……..... ○ 1

Female ….... ○ 2

Diverse ……... ○ 3

PHYSICAL ACTIVITIES DURING SCHOOL TIME

The following questions deal with your child's physical activities at school. When answering the following questions, **please think of the last 7 days of school.** If your child has holidays right now or is sick, just think about his/her last complete school week.

1. How far is your child's school located from your home?
Please select only one answer.

Less than 1 km	○ 1
1 – 2 km	○ 2
3 – 4 km	○ 3
5 – 6 km	○ 4
More than 6 km	○ 5

2. How does your child usually get to or from school?
Please tick only one answer for the "travel to" and only one answer for the "travel home".

**Travel to school:**		**Travel home:**	
Walking	○ 1	Walking	○ 1
Cycling, skating, or non-motorized scooters	○ 2	Cycling, skating, or non-motorized scooters	○ 2
School bus and/or public transportation	○ 3	School bus and/or public transportation	○ 3
By car, scooter, motorcycle	○ 4	By car, scooter, motorcycle	○ 4

3. How much time of physical education does your child have in total in a <u>typical school week?</u>
Please give the total in hours and minutes for a whole week.

☐☐ hour(s) and ☐☐ minutes per school week

None .... ○ 1

Don't know .... ○ 7

4. How long are your child's school breaks in total on a typical school day?
Please give the total in hours and minutes of short and long breaks for an entire school day.

☐☐ hour(s) and ☐☐ minutes per day

Don't know .... ○ 7

PHYSICAL ACTIVITIES DURING YOUR CHILD'S LEISURE TIME

The following questions are about your child's physical activities that he/she does solely in his/her leisure time. Please do not include any activities that you have already mentioned! While answering the following questions, **<u>please think about the last month.</u>**

<u>On weekdays after school</u>

5. How much time does your child spend playing outdoors <u>on a typical school day in his/her leisure time</u>?

☐☐ hour(s) and ☐☐ minutes outdoors per day

None .... ○ 1 ⇨ Please continue with question 6

How intensive is the physical activity of your child when playing outside?
Please select only one answer.

Not sweating and not out of breath	○ 1
Slightly sweating and a little out of breath	○ 2
Sweating a lot and very out of breath	○ 3
Don't know	○ 7

On weekend days

6. How much time does your child spend playing outdoors on a typical weekend day?

☐☐ hour(s) and ☐☐ minutes outdoors per weekend day

None ……. ○ 1 ⇨ Please continue with question 7

How intensive is the physical activity of your child when playing outside?
Please select only one answer.

Not sweating and not out of breath	○ 1
Slightly sweating and a little out of breath	○ 2
Sweating a lot and very out of breaths	○ 3
Don't know	○ 7

Sports club

7. Is your child member of a sports club? *Please select only one answer.*

Yes ……. ○ 1

No ……. ○ 2 ⇨ Please continue with question 9

8. What kind of sport does your child do in a sports club?
Please tick all answers that apply. Multiple answers are possible.

Soccer	○ 1
Other ball sports (e.g. basketball, volleyball, handball, field/ ice hockey)	○ 1
Racquet sports (e.g. tennis, badminton, table tennis)	○ 1
Dancing (incl. figure skating)	○ 1
Gymnastics (incl. rhythmic gymnastics)	○ 1
Martial arts (e.g. judo, karate, boxing, win tsun)	○ 1
Athletics	○ 1
Swimming	○ 1
Other endurance sports (e.g. aerobics, cycling, triathlon, nordic walking, running).	○ 1
Strength training (e.g. in a fitness studio)	○ 1
Health training (e.g. yoga, pilates)	○ 1
Other ⇨ Please specify: ______________________	○ 1

SLEEPING HABITS OF YOUR CHILD

Now we would like to know about the typical sleeping habits of your child during school days and during the weekends/vacations.

Your child's sleep duration on school days

9. When does your child usually go to bed on school days?

Please enter the time. An example: If your child usually goes to bed at seven thirty in the evening, enter 1 9 : 3 0

at ☐☐ : ☐☐

10. When does your child usually wake up on school days?

Please enter the time. An example: If your child usually wakes up at six in the morning, enter 0 6 : 0 0

at ☐☐ : ☐☐

Your child's sleep duration during the weekends/holidays

11. When does your child usually go to bed on weekends/vacation?

Please fill in the time. Example: If your child normally goes to bed at seven thirty at night, enter

1 9 : 3 0

at ☐☐ : ☐☐

12. When does your child usually wake up on weekends/vacation?

Please fill in the time. Example: If your child normally wakes up at six in the morning, enter

0 6 : 0 0

at ☐☐ : ☐☐

13. Please answer the following questions on your child's typical sleeping habits and his/her condition during the daytime. *Please tick one answer per line*.

	Yes	No
Does your child have a regular bedtime routine?	○ 1	○ 2
Does your child have difficulty to fall asleep?	○ 1	○ 2
Does your child have trouble getting up in the morning?	○ 1	○ 2
Is your child sleepy during the day?	○ 1	○ 2

DIET QUESTIONNAIRE

14. How often does your child usually eat?
Please tick one answer per line.

	Never	On fewer occasions than once a week	1-2 times per week	3-6 times per week	Daily
Breakfast	○ 1	○ 2	○ 3	○ 4	○ 5
Morning snack(s)	○ 1	○ 2	○ 3	○ 4	○ 5
Lunch	○ 1	○ 2	○ 3	○ 4	○ 5
Afternoon snack(s)	○ 1	○ 2	○ 3	○ 4	○ 5
Dinner	○ 1	○ 2	○ 3	○ 4	○ 5
Evening snack(s)	○ 1	○ 2	○ 3	○ 4	○ 5

15. During the <u>last seven days</u>, how often did most of the family eat a meal together? *Please select only one answer.*

Never/less than once a week	1-3 times a week	4-6 times a week	Once a day	Twice a day or more
○ 1	○ 2	○ 3	○ 4	○ 5

16. How often does your child eat while doing something else (e.g. watching TV, playing, using a smartphone, iPad or tablet…)?
Please tick only one answer.

Never or rarely	Several times per week	Once a day	On several occasions per day
○ 1	○ 2	○ 3	○ 4

17. How many times does your child eat in a fast food restaurant (e.g., McDonalds, Burger King) or stands or kiosks (e.g., kebab, sausage stand, French fry stand, Asian snack bar)? *Please tick one answer per line.*

Never	Once a month or less	Several times a month	1-2 times a week	3 or more times a week
○ 1	○ 2	○ 3	○ 4	○ 5

18. Do you think your child is… *Please select only one answer.*

much too underweight? …………………………… ○ 1

slightly too underweight? ……………………… ○ 2

proper weight? …………………………………… ○ 3

slightly too overweight? ………………………… ○ 4

much too overweight? …………………………… ○ 5

19. Over a typical or usual week, how often does your child eat or drink the following kinds of foods or beverages? *Please tick one box for each line.*

	Never	Less than once a week	On some days (1-3 days)	Most days (4-6 days)	Every day
Fresh fruit	○ 1	○ 2	○ 3	○ 4	○ 5
Vegetables (including vegetable soup, except potatoes)	○ 1	○ 2	○ 3	○ 4	○ 5
Sugary soft drinks	○ 1	○ 2	○ 3	○ 4	○ 5
Breakfast cereal	○ 1	○ 2	○ 3	○ 4	○ 5
Please read the nutrition label and check quantity/content of: carbohydrates: _______ g/100g; sugar: _______ g/100g					
Meat	○ 1	○ 2	○ 3	○ 4	○ 5
Fish	○ 1	○ 2	○ 3	○ 4	○ 5
Egg dishes	○ 1	○ 2	○ 3	○ 4	○ 5
Low fat/ semi-skimmed milk	○ 1	○ 2	○ 3	○ 4	○ 5
Whole-fat milk	○ 1	○ 2	○ 3	○ 4	○ 5
Flavoured milk	○ 1	○ 2	○ 3	○ 4	○ 5

	Never	Less than once a week	On some days (1-3 days)	Most days (4-6 days)	Every day
Cheese	◯ 1	◯ 2	◯ 3	◯ 4	◯ 5
Yogurt, milk pudding, cream cheese/quark or other dairy products	◯ 1	◯ 2	◯ 3	◯ 4	◯ 5
100% fruit juice	◯ 1	◯ 2	◯ 3	◯ 4	◯ 5
Diet or "light" soft drinks	◯ 1	◯ 2	◯ 3	◯ 4	◯ 5
Savoury snacks (e.g. potato chips, corn chips, popcorn, peanuts)	◯ 1	◯ 2	◯ 3	◯ 4	◯ 5
Sweet snacks (e.g., cakes, cookies, sweets)	◯ 1	◯ 2	◯ 3	◯ 4	◯ 5
Legumes (e.g. beans, lentils)	◯ 1	◯ 2	◯ 3	◯ 4	◯ 5

MEDIA CONSUMPTION AND MEDIA USE

Now we would like to know how long your child uses the different media and what devices he or she is currently using.

20. How long does your child usually watch television and/ or movies/DVD per day?
Please tick one answer per line.

	Not at all	Less than 30 minutes daily	Between 30 minutes and 2 hours a day	Between 2 and 3 hours a day	Between 3 and 6 hours a day	More than 6 hours per day
On weekdays	◯ 0	◯ 1	◯ 2	◯ 3	◯ 4	◯ 5
On weekends	◯ 0	◯ 1	◯ 2	◯ 3	◯ 4	◯ 5

21. How long does your child usually play electronic games (at a computer, game console, smartphone, iPad, etc.) per day? *Please tick one answer per line.*

	Not at all	Less than 30 minutes daily	Between 30 minutes and 2 hours a day	Between 2 and 3 hours a day	Between 3 and 6 hours a day	More than 6 hours per day
On weekdays	◯ 0	◯ 1	◯ 2	◯ 3	◯ 4	◯ 5
On weekends	◯ 0	◯ 1	◯ 2	◯ 3	◯ 4	◯ 5

22. How often does your child use a smartphone on a typical day?
Please tick only one answer.

- It does not have access to a smartphone ………..… ◯1
- Less than 5 times per day …………………………..…. ◯2
- 6 – 10 times per day………………………………….…… ◯3
- 11 – 20 times per day ……………………………………… ◯4
- 21 – 50 times per day ………………………….…….…… ◯5
- 51 – 100 times per day ……………………………….……. ◯6
- More than 100 times per day ……………………..…..…. ◯7

STRENGTHS AND DIFFICULTIES OF YOUR CHILD

23. Strenghts and difficulties
For each item, please mark the box for Not True, Somewhat True or Certainly True. It would help us if you answered all items as best you can even if you are not absolutely certain or the item seems daft! Please give your answers on the basis of the child's behaviour *<u>over the last six months</u>* *or this school year.*
Please mark one answer per line.

	Not True	Somewhat True	Certainly True
Considerate of other people's feelings	◯ 1	◯ 2	◯ 3
Restless, overactive, cannot stay still for long	◯ 1	◯ 2	◯ 3
Often complains of headaches, stomach-aches or sickness	◯ 1	◯ 2	◯ 3
Shares readily with other children (treats, toys, pencils etc.)	◯ 1	◯ 2	◯ 3
Often loses temper	◯ 1	◯ 2	◯ 3
Rather solitary, tends to play alone	◯ 1	◯ 2	◯ 3
Generally obedient, usually does what adults request	◯ 1	◯ 2	◯ 3
Many worries, often seems worried	◯ 1	◯ 2	◯ 3

	Not True	Somewhat True	Certainly True
Helpful if someone is hurt, upset or feeling ill	○ 1	○ 2	○ 3
Constantly fidgeting or squirming	○ 1	○ 2	○ 3
Has at least one good friend	○ 1	○ 2	○ 3
Often fights with other children or bullies them	○ 1	○ 2	○ 3
Often unhappy, depressed or tearful	○ 1	○ 2	○ 3
Generally liked by other children	○ 1	○ 2	○ 3
Easily distracted, concentration wanders	○ 1	○ 2	○ 3
Nervous or clingy in new situations, easily loses confidence	○ 1	○ 2	○ 3
Kind to younger children	○ 1	○ 2	○ 3
Often lies or cheats	○ 1	○ 2	○ 3
Picked on or bullied by other children	○ 1	○ 2	○ 3
Often offers help to others (parents, teachers, other children)	○ 1	○ 2	○ 3
Thinks things out before acting	○ 1	○ 2	○ 3
Steals at home, school or elsewhere	○ 1	○ 2	○ 3
Gets along better with adults than with other children	○ 1	○ 2	○ 3
Many fears, easily scared	○ 1	○ 2	○ 3
Good attention span, sees work through to the end	○ 1	○ 2	○ 3

Thank you for answering these questions!

Field work notes:
