# Supplementary material for: Implementing a family-based intervention to promote healthy family routines in deprived neighborhoods – a feasibility study from Bremen, Germany
Source: BMC Public Health. 2025 Dec 23;25:4344. doi: 10.1186/s12889-025-25532-9 (PMC12751738; doi:10.1186/s12889-025-25532-9)
Supplement: Supplementary file 7 — Supplementary Material 7. [file 12889_2025_25532_MOESM7_ESM.docx]

| **RE-AIM criteria** | **Theme** | **Description** | **Codes** | **Examples of quotes** (referring to codes in bold) |
| --- | --- | --- | --- | --- |
| Maintenance | Ensuring recruitment in the long-term | Stakeholders consider certain aspects to be meaningful and crucial for the target group of socially disadvantaged families to consider such a program and to register for it | **# Integration of the intervention within trustworthy institutions**  **# Recruitment via trusted contact persons**  # Comprehensible program advertisement  # Fixed collaborations with other local stakeholders | „When we try to refer them [the families] elsewhere, this is often a great barrier. So, if you connect such a program to a kindergarten or a family centre, I believe the success is greater because I simply think they need a trusting environment that they know, not having to meet someone new again, not having to go to someone else, and not knowing what to expect.” [Coordinator of a local kindergarten, focus group discussion with stakeholders after the intervention] |
|  | Increasing the attractiveness of the program in the long term | To ensure that the program is also sought after in the long term by the targeted families, certain points in program design and implementation should be considered from the perspective of stakeholders | **# Adding more practical intervention elements**  # Sensitive, non-intrusive approach regarding home visits  # Resources for childcare during visits  # (Language) diversity of health educators | „And we have also noticed that when we offer practical things, they [the families] are more likely to participate. If we were to actually cook or set up a stand for healthy eating, for example, that would be more helpful than just providing advice.” |
|  | Fostering long-term effectiveness | Factors that contribute to the long-term effectiveness of the program, so that the learned health behaviors are continued on a family level even after the program ends | **# Opportunities for networking among the families after intervention**  # Integrated community-wide approach  # Strategic coordination of existing health promotion activities | „… with the four visits, for example, to check, is that enough now? Or do we leave the family somewhere and maybe need them to access a website or something where they can refer back to…” |

**Additional file 7: Coding frame example**
